# Fatal Illness and Dissection of Dr. Gall

**Published:** 1829

**Authors:** 


40. *Fatal Illness and Dissection of Dr. Gall.*—Believing that most of our readers, like ourselves, feel a laudable curiosity to know something of the last illness and *post mortem* appearances, of men distinguished in the profession; we shall extract from Johnson's Review for January, 1829, a condensed account of this kind, relative to the celebrated Dr. Gall, whose death we announced in a former number of this Journal.

Dr. Gall was above the middle stature, having an expanded chest, strong muscular extremities—large head—high forehead—open and pleasing countenance. Endowed with a most vigorous constitution, he was able to dedicate a long life to deep and almost incessant study, with trifling inconvenience to his bodily health. Two or three attacks of the gout, and the same of gastro-intestinal disorder, were all that he experienced till of late years, when his gait became heavy, and his breathing short, while ascending any eminence, or even his stairs. To this was added palpitation. About eighteen months preceding his death, these symptoms had increased, and obliged Dr. G. to keep very quiet, to live low, and lose blood several times.—Messrs. Rostan, Andral, Dennisi, and Robouam, examined Dr. Gall's chest repeatedly by the stethoscope, and recognized hypertrophy with dilation of the heart—especially of the left ventricle. By the means above-mentioned, Dr. G. got something better, and resumed his usual occupations. In November last he commenced his course of lectures at the Athenæum, and continued them till April of 1828. On the third of that month, he experienced some symptoms of cerebral congestion—and on the 20th of the same month, there was incomplete hemiplegia of the right side, which remained in despite of the usual means. In the month of May, the Doctor had recourse to some active purgatives, which produced a prolapsus of the rectum, and hæmorrhoidal tumors. Electricity, acupuncturation, and other means having failed, Dr. G. determined on change of air and repaired to Montrouge. Here he continued to be affected with nausea, loss of appetite, &c. and took an emetic which operated upwards and downwards, and brought on a slight attack of gout. But the redness and dryness of the tongue, and the irritability of the stomach, clearly showed that the digestive apparatus was the seat of disease, and M. Broussais was added to the list of consultants. This gentleman gave an unfavorable prognosis, which was but too soon verified. It was remarked—and was certainly very remarkable, that throughout the whole of these attacks, even that of gout, there was no symptom of re-action, or of febrile movement. On the 6th of August, Dr. G. experienced a chill, which was not succeeded by any re-action. Next day, (7th) there was a violent rigor of an hour's duration, and this was followed by some febrile movement, and ultimately by perspiration. The patient now insisted on taking sulphate of quinine, which he took to the amount of twenty grains in twenty-four hours. Dr. G. still preserved the full possession of his intellectual faculties, and his countenance presented a natural aspect. The pulse, which had been irregular, now ceased to intermit. He was able to take chicken and chicken broth with ease. During the next four or five days, the quinine was continued, at the rate of 24 grains a day. The patient ate and digested each day—but now the cheeks began to exhibit some flush, the eyes altered their expression, and the ideas wandered. On the 14th, the tonic plan was changed for the soothing, as the head was evidently affected. He now sunk into a comatose state, from which he never emerged. On the 21st of August, Dr. Gall paid the last debt of Nature!

*Post-mortem examination.*—The head was very large, as we before observed—the cranium was remarkably thick, at least double the natural thickness. The channels of the dura matral arteries were remarkably deep—pia mater infiltrated with serosity, and several ounces of water were found at the base of the skull. The brain was not dissected, but preserved in spirits. It weighed two pounds ten ounces and a quarter, French—little under three pounds English. There was a small tumour near the surface of the cerebellum.—The interior condition of the brain could not, of course, be ascertained In the chest, the lungs were found to be perfectly healthy—heart one-third larger than natural, being flabby in texture, and containing a considerable quantity of blood. The chambers were larger than natural, and their parietes thickened, especially the left ventricle. The arch of the aorta was dilated, and the internal surfaces of most of the large vessels were red, not the effects of imbibition. The parieties of the stomach were generally thickened, and the mucous membrane red, soft, hypertrophied, and, in some places eroded. The lining of the duodenum, and small intestines was nearly in a similar state. The gall-bladder contained numerous calculi, and was divided into two pouches quite distinct.

Such are the particulars of Dr. Gall's death. It is evident that he did not die of the affection of the heart. Whether the large quantities of sulphate of quinine, taken when the mucous membrane of the stomach and bowels was in a state of inflammation, may have accelerated Dr. Gall's death, by determining to the head, admits of doubt—or rather it admits of little doubt.

